# Identification of Piezo1 as a potential target for therapy of colon cancer stem-like cells

**DOI:** 10.1007/s12672-023-00712-4

**Published:** 2023-06-12

**Authors:** Rong Li, Dongmei Wang, Huijuan Li, Xianhua Lei, Weilian Liao, Xiao-Yu Liu

**Affiliations:** 1 Department of Pathology, Ganzhou Cancer Hospital, No. 19, HuaYuan Qian Road, Ganzhou, Jiangxi China; 2 School of Medicine, Southern University of Science and Technology, 1088 Xueyuan Avenue, Shenzhen, Guangdong China

**Keywords:** Colon cancer stem-like cells, Piezo1, NFAT1, Calcium, Stemness

## Abstract

Colon cancer is a common malignancy of the digestive tract. Colon cancer stem-like cells (CCSCs) are theoretically one of the key drivers of the initiation, relapse, metastasis, and chemo-resistance of colon tumors. Piezo1 is a mechanosensitive cationic channel protein involved in cancer progression. However, little is known regarding the possible role of Piezo1 in maintaining the stemness of CCSCs. In this study, we found that Piezo1 was highly expressed in CD133^+^/CD44^+^ colon cancer tissues, and the Piezo1^high^/CD133^+^CD44^+^ population was associated with the clinical stage. Furthermore, CCSCs isolated from colon cell lines expressed higher Piezo1 levels compared to the non-CCSCs, and Piezo1 knockdown inhibited their tumorigenicity and self-renewal capacity. Mechanistically, Piezo1 maintained the stemness of CCSCs through Ca^2+^/NFAT1 signaling, and knocking down Piezo1 promoted degradation of NFAT1. Taken together, Piezo1 is involved in the stage of colon cancer and is a promising therapeutic target.

**Supplementary Information:**

The online version contains supplementary material available at 10.1007/s12672-023-00712-4.

## Introduction

Colon cancer is the second most common malignancy worldwide and the fourth leading cause of cancer-related deaths [1]. More than 1.2 million new cases of colon cancer are detected every year, and about 600,000 deaths occur due to colorectal cancer annually [2]. With improvements in living standards and changes in dietary habits, the incidence of colon cancer has increased significantly in Asian counties in recent years [3]. The early stage of colon cancer is often asymptomatic, and most patients are in the middle or late stage of the disease when diagnosed. In addition, the 5-year survival rate after surgery is still low [4], and there is a lack of targeted therapies for colon cancer. Therefore, it is crucial to elucidate the mechanisms underlying the development and progression of colon cancer. Studies increasingly show that colon cancer cells are heterogenous in terms of their morphology and functions. A subpopulation of cancer cells, termed colon cancer stem-like cells (CCSCs), has been identified that exhibits self-renewal and multipotent differentiation ability. There is evidence that CCSCs are key determinants of tumor initiation, relapse, and metastasis due to their ability to remain quiescent, renew and terminally differentiate [5, 6]. Therefore, specifically targeting the CCSCs may lead to complete tumor clearance, thereby reducing the risk of recurrence and improving patient survival [7]. However, the molecular mechanisms underlying the development and functions of CCSCs are not entirely clear, and need to be elucidated in order to develop novel therapeutic strategies for colon cancer.

Intracellular calcium levels control integral cellular processes, such differentiation, survival, death, and movement under physiological or pathological conditions [8]. Furthermore, abnormal expression and function of calcium channels have been linked to tumorigenesis [9]. Coste et al. discovered the mechanosensitive ion channel protein Piezo1, also known as FAM38A, which is a subunit of mechanically activated cation channels [10]. Piezo1 is widely expressed in human organs and tissues, including stomach, colon, brain, lung, heart, bone, cardiovascular system, and immune system [11]. Recent studies have shown that Piezo1 plays a critical role in the proliferation, migration, and differentiation of multiple cancer cells [12]. Zhang et al. found that Piezo1 is overexpressed in gastric cancer cells and correlates with poor survival rate. Moreover, Piezo1 may promote cell proliferation and invasion by accelerating cell cycle transition [13]. YAP promotes proliferation of oral squamous carcinoma cells by upregulating Piezo1 through activation of the Hippo pathway [13]. Furthermore, Piezo1 is known to mediate prostate cancer cell proliferation and migration via AKT/mTOR signaling [14]. It is highly expressed in colon cancer tissues and is associated with poor prognosis of patients. At the molecular level, Piezo1 promotes colon cancer cell migration and invasion via MCU/HIF-1α/VEGF signaling [15]. A recent study showed that Piezo1-mediated calcium signaling may affect the migration of human endometrial mesenchymal stem cells [16]. In addition, Piezo1 is upregulated in periosteal stem cells [17], and also determines the fate of neural stem cells during brain development by regulating cholesterol biosynthesis [18]. However, the possible function of Piezo1 in maintaining the phenotype of CCSCs is unclear.

Given that the role of CCSCs in the relapse and chemo-resistance of colon tumor cells, and the critical role of Piezo1 in cancer development, we hypothesized that Piezo1 may regulate the stemness and tumorigenic potential of CCSCs. In this study, we found that Piezo1 knockdown impaired the stemness of CCSCs in vitro and in vivo, which suggests that Piezo1 is a potential therapeutic target for colon cancer.

## Materials and methods

### Tissue samples

The present study was approved by the Ethical Committee of Ganzhou Cancer Hospital (2020018), and written informed consent was obtained from all participants. The study was performed in accordance with the principles of the Declaration of Helsinki regarding research involving human subjects.

### Cell lines and culture

The human colon cancer cell lines SW480 and HCT-8 were purchased from American Type Culture Collection. HCT-116 and HT-29 were purchased form the Chinese Academy of Sciences Cell Bank. The cells were cultured in McCoy’s 5 A or Leibovitz’s L-15 medium supplemented with 10% FBS, 100 U/mL penicillin and 100 µg/mL streptomycin at 37 °C in a humidified incubator with 95% O_2_ and 5% CO_2_.

### Isolation of CCSCs

CD133^+^CD44^+^ cells were isolated by a two-step magnetic cell sorting method as previously described [19]. Briefly, the cells were incubated with CD44 MicroBeads (Miltenyi Biotec) and the CD44^+^ fraction was isolated using the autoMACS™ Pro separator (Miltenyi Biotec). Following incubation with CD133 MicroBeads (Miltenyi Biotec), the CD44^+^CD133^+^ cells were isolated as above.

### siRNA transfection

The cells were transfected with siRNAs using DharmaFECT 1 Transfection Reagent (GE Dharmacon) according to the manufacturer’s instructions. The siRNA sequences were as follows: siPiezo1#1: 5′-CUGAUGUUGUCGACUUCAUtt-3′, siPiezo1#2: 5′-CCAUCCACCUAUGGAUGUUtt-3′, siPiezo1#3: 5′-GAGAUCUCGCACUCCAUUAtt-3′. NTAT1 siRNA(h) was purchased form Santa Cruz Biotechnology, Inc.

### Real-time RT-PCR

Total RNA was isolated using the RNeasy Mini Kit (Qiagen) according to the manufacturer’s instructions. RT-PCR was performed using Superscript II reverse transcriptase (Invitrogen) following the manufacturer’s instructions. Real-time PCR was performed using QuantiNova SYBR Green PCR Master Mix (Qiagen) on the Applied Biosystems 7500 FAST Real Time PCR system. β-actin was used as an internal control. Each reaction was carried out in triplicate. The primer sequences were as follows: β-actin forward 5′-CACCATTGGCAATGAGCGGTTC-3′, reverse 5′-AGGTCTTTGCGGATGTCCACGT-3′; Piezo1 forward 5′-CCTGGAGAAGACTGACGGCTAC-3′, reverse 5′-ATGCTCCTTGGATGGTGAGTCC-3′; NFAT1 forward 5′-GATAGTGGGCAACACCAAAGTCC-3′, reverse 5′-TCTCGCCTTTCCGCAGCTCAAT-3′; Nanog forward 5′-CTCCAACATCCTGAACCTCAGC-3′, reverse 5′-CGTCACACCATTGCTATTCTTCG-3′; Sox2 forward 5′-GCTACAGCATGATGCAGGACCA-3′, reverse 5′-TCTGCGAGCTGGTCATGGAGTT-3′; Oct4 forward 5′-CCTGAAGCAGAAGAGGATCACC-3′, reverse 5′-AAAGCGGCAGATGGTCGTTTGG-3′.

### Western blotting

Western blotting was performed as previously described [20]. The blots were incubated overnight with primary antibodies targeting β-actin (Santa Cruz), Piezo1 (Poteintech), NFAT1 (Santa Cruz), NFAT2 (Santa Cruz), NFAT3 (Santa Cruz), NFAT4 (Santa Cruz), and TBP (Abcam) at 4 °C with constant shaking. After incubating with HRP-labeled secondary antibodies (Cell Signaling) at room temperature for 2 h, the protein bands were detected using the Pierce™ ECL Western Blotting Substrate (Thermo Scientific).

### Immunohistochemistry

Immunohistochemistry was performed as previously described [21]. Tissue slides were incubated with anti-Piezo1 (1:200; Proteintech), anti-CD133 (1:200; Proteintech), or anti-CD44 (1:200; Proteintech) antibodies at 4 °C in a humidified chamber. The color was developed using the GTVision III Detection System/Mo&Rb Kit (Gene Tech). The widely accepted German semi-quantitative scoring system was used to assess the staining intensity and proportion of stained cells. Each specimen was assigned a score according to the intensity of staining (0, none; 1, weak; 2, moderate; 3, strong) and the proportion of stained cells (0, 0%; 1, 1–24%; 2, 25–49%; 3, 50–74%; 4, 75–100%). The final immune reactive score was determined by multiplying the intensity score by the percentage score, ranging from 0 to 12.

### Cell growth assay

The cells were seeded in 96 well plates, cultured for 7 days and incubated with 10 µL/well Cell Counting Kit-8 reagent (MedChemExpress HK-K0301) for 2 h at 37 °C. The absorbance was measured at 450 nm using a multi-mode plate reader (Molecular Devices).

### Clonogenic assay

Clonogenic assay was performed as described previously [16]. Briefly, the cells were seeded in a 6-well plate at the density of 200 cells/well and cultured for 10–12 days. The colonies were fixed with 4% paraformaldehyde for 15 min and stained with crystal violet. Colonies with more than 50 cells were counted.

### Sphere formation assay

Single cells were seeded in ultra-low attachment plates (Corning) at the density of 1000 cells/well in serum-free DMEM/F12 medium (Invitrogen) supplemented with B27 (Invitrogen), 20 ng/mL EGF and 10 ng/mL bFGF (Peprotech). After 10 days, plates were analyzed and quantified using an inverted light microscope (Nikon).

### Ca^2+^ imaging

Briefly, cells were loaded with 0.02% pluronic F-127 (Invitrogen) and 10 µM Fluo-4/AM (Invitrogen) at 37 °C for 30 min in the dark. After washing thrice with normal physiological saline solution (NPSS, 140 mmol/L NaCl, 5 mmol/L KCl, 1 mmol/L CaCl_2_, 1 mmol/L MgCl_2_, 10 mmol/L glucose, and 5 mmol/L HEPES, pH 7.4), the cells were observed under a Nikon A1R confocal microscope. The ratio of the initial to final fluorescence signals (F1/F0) were calculated to quantify the change in cytosolic Ca^2+^ levels.

### Xenograft tumor model

Tumor cells were suspended in 50 µL PBS and 50 µL Matrigel (BD Biosciences) and injected subcutaneously into nude mice (male) [21, 22]. Tumor growth was observed every week. Mice with no tumor burden were generally terminated 10 weeks after tumor cell injection after confirming lack of tumor development. All experiments were conducted according to the Guide for Animal Care and Use of Laboratory Animals published by the US National Institute of Health, and approved by the Ethics Committee of Ganzhou Cancer Hospital.

### Statistical analysis

Statistical analysis was performed using GraphPad Prism. Data were expressed as mean ± standard deviation (SD) of at least three independent experiments. Data was compared using Student’s t-test or one-way ANOVA as appropriate. *P* value < 0.05 was considered statistically significant.

## Results

### Piezo1 is positively associated with CCSCs

**Fig. 1:**
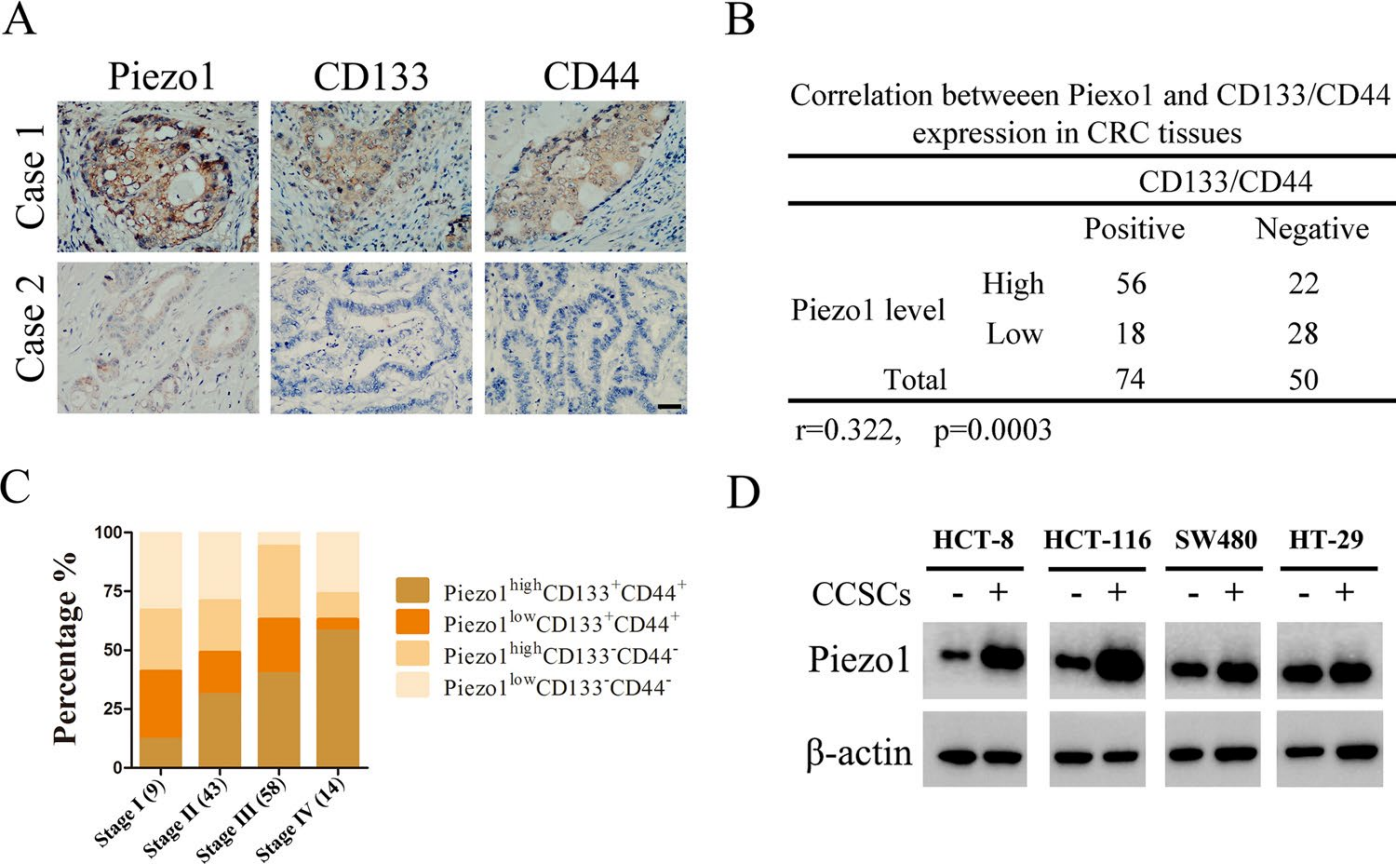
Piezo1 expression in CD133^+^CD44^+^ colon cancer tissues. **A** Immunohistochemical images showing in-situ expression of Piezo1, CD133 and CD44 in colon cancer tissues. Scale bar = 20 μm. **B** Correlation between Piezo1 and CD133/CD44 expression in colon cancer tissues. **C** The proportion of Piezo1^high^/CD133^+^CD44^+^ patients in advanced stage of colon cancer. **D** Immunoblot showing Piezo1 expression in CCSCs (CD133^+^CD44^+^) isolated from colon cancer cell lines

CCSCs are identified on the basis of the surface expression of CD133 and CD44 [23]. To assess the relationship between Piezo1 and CCSCs in colon cancer patients, we evaluated the expression of Piezo1, CD133 and CD44 in the tumor tissues. As shown in Fig. 1A, B, Piezo1 expression was higher in CD133^+^/CD44^+^ tissues and correlated positively with CD133/CD44 expression. Accordingly, we classified the patients into Piezo1^high^/CD133^+^CD44^+^, Piezo1^low^/CD133^+^CD44^+^, Piezo1^high^/CD133^−^CD44^−^, and Piezo1^low^/CD133^−^CD44^−^ groups. The Piezo1^high^/CD133^+^CD44^+^ patients were usually in the advanced clinical stages, suggesting poor prognosis (Fig. 1C). Consistent with the in-situ results, Piezo1 was overexpressed in the CD133^+^CD44^+^ fraction of multiple colon cancer cell lines, including HCT-116, HCT-8, SW480 and HT-29 (Fig. 1D; Supplementary Figure 1). Taken together, Piezo1 expression is closely related to CCSCs and the stage of colon cancer patients.

### Piezo1 knockdown impaired the tumorigenic potential and stemness of the CCSCs

**Fig. 2:**
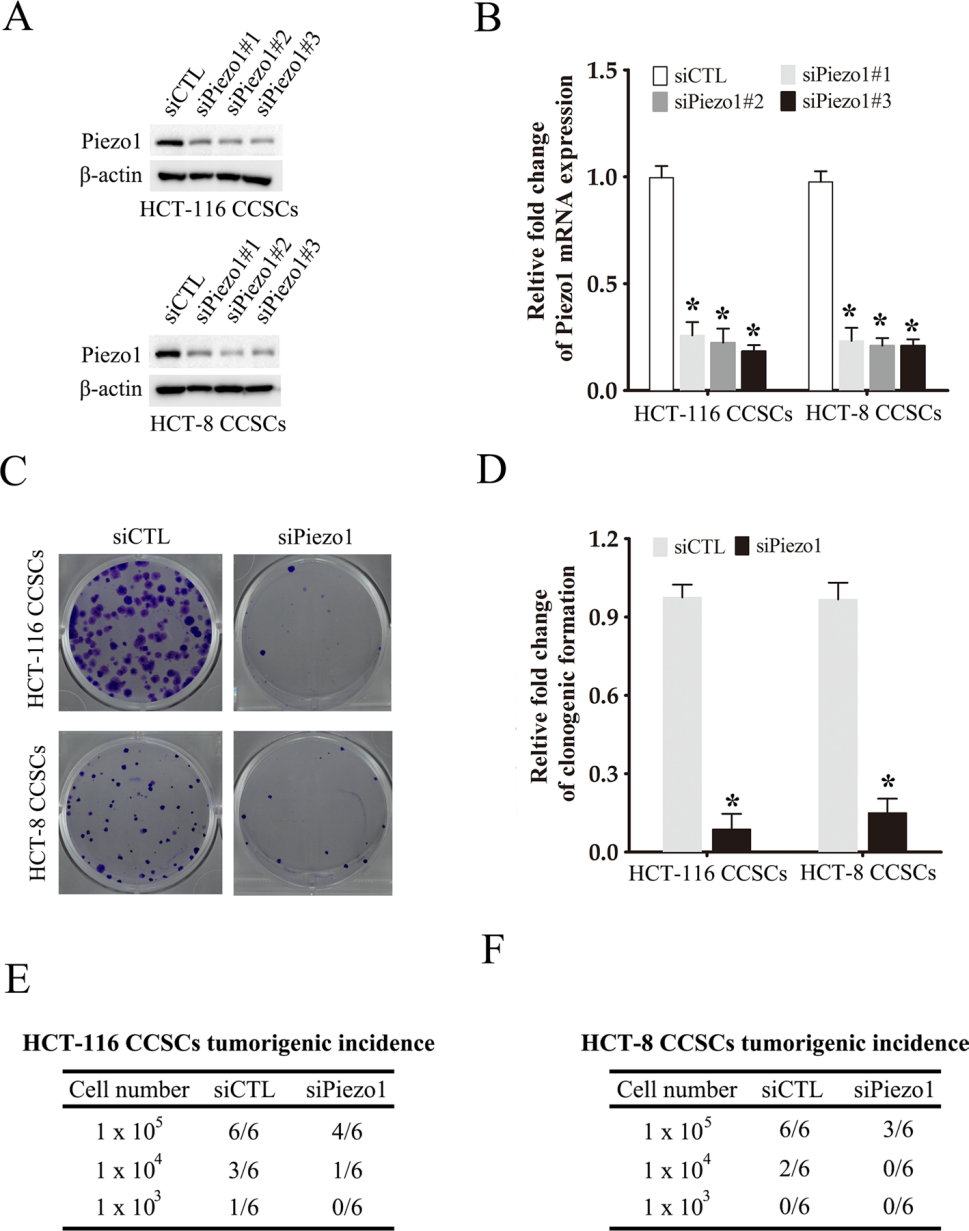
Piezo1 knockdown inhibited tumorigenicity of CCSCs. **A**, **B** Piezo1 protein and RNA expression in Piezo1-knockdown CCSCs. The data represent the mean ± SD. *p < 0.05, versus siCTL. **C**, **D** Representative images and number of colonies formed by CCSCs transfected with siPiezo1 or siCTL. The data represent the mean ± SD. *p < 0.05, versus siCTL. **E**, **F** Xenografts formed by CCSCs with Piezo1 knockdown

**Fig. 3:**
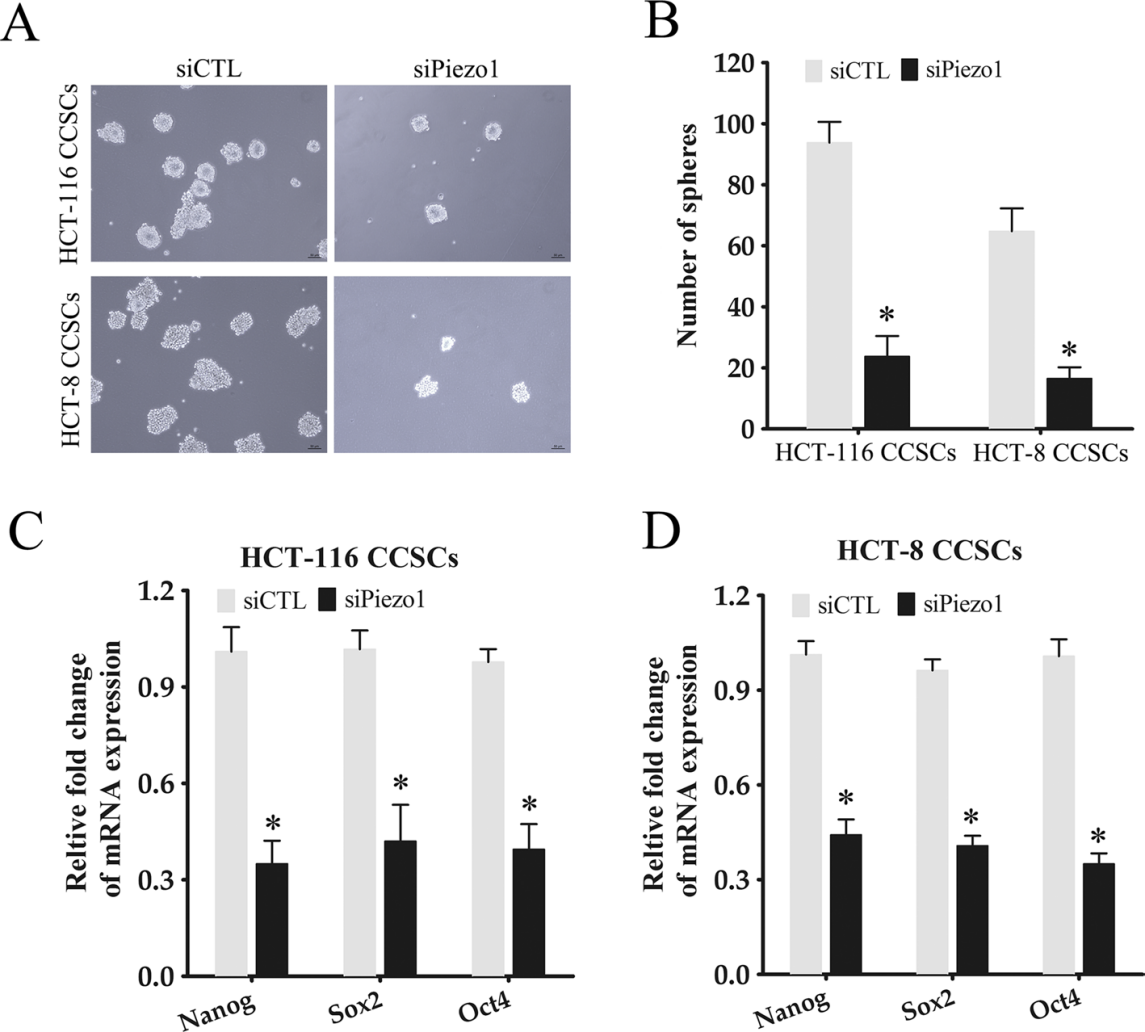
Piezo1 knockdown inhibited stemness of CCSCs. **A**, **B** Representative images and number of spheres formed by CCSCs transfected with siPiezo1 or siCTL. The data represent the mean ± SD. *p < 0.05, versus siCTL. **C**, **D** Nanog, Sox2 and Oct4 mRNA levels in CCSCs transfected with siPiezo1 or siCTL. The data represent the mean ± SD. *p < 0.05, versus siCTL

To further explore the biological function of Piezo1 in CCSCs, we knocked down the gene in the CCSCs derived from HCT-116 and HCT-8 cells using three specific siRNAs. Piezo1 knockdown in the CCSCs significantly decreased its protein (Fig. 2A) and mRNA (Fig. 2B) levels. Knocking down Piezo1 significantly decreased the number of colonies and cell growth formed by the CCSCs compared to the respective control cells (Fig. 2C, D; Supplementary Figure S2). Furthermore, we established a subcutaneous xenograft tumor model using CCSCs to further characterize the tumorigenic role of Piezo1 in vivo. Piezo1 knockdown markedly inhibited tumor formation by the CCSCs compared to that in the control groups (Fig. 2E, F). The self-renewal capacity of the CCSCs was evaluated by the sphere formation assay. As shown in Fig. 3A, B, downregulation of Piezo1 in the CCSCs significantly decreased their ability to form spheres in serum-free conditions. Consistent with this, the stemness-related genes Nanog, Sox2 and Oct4 were significantly downregulated in the Piezo1-knockdown CCSCs compared to the control group (Fig. 3C, D). Taken together, Piezo1 knockdown impaired the stem-like characteristics of CCSCs.

### Piezo1 overexpression enhanced the tumorigenic potential and stemness of CCSCs

**Fig. 4:**
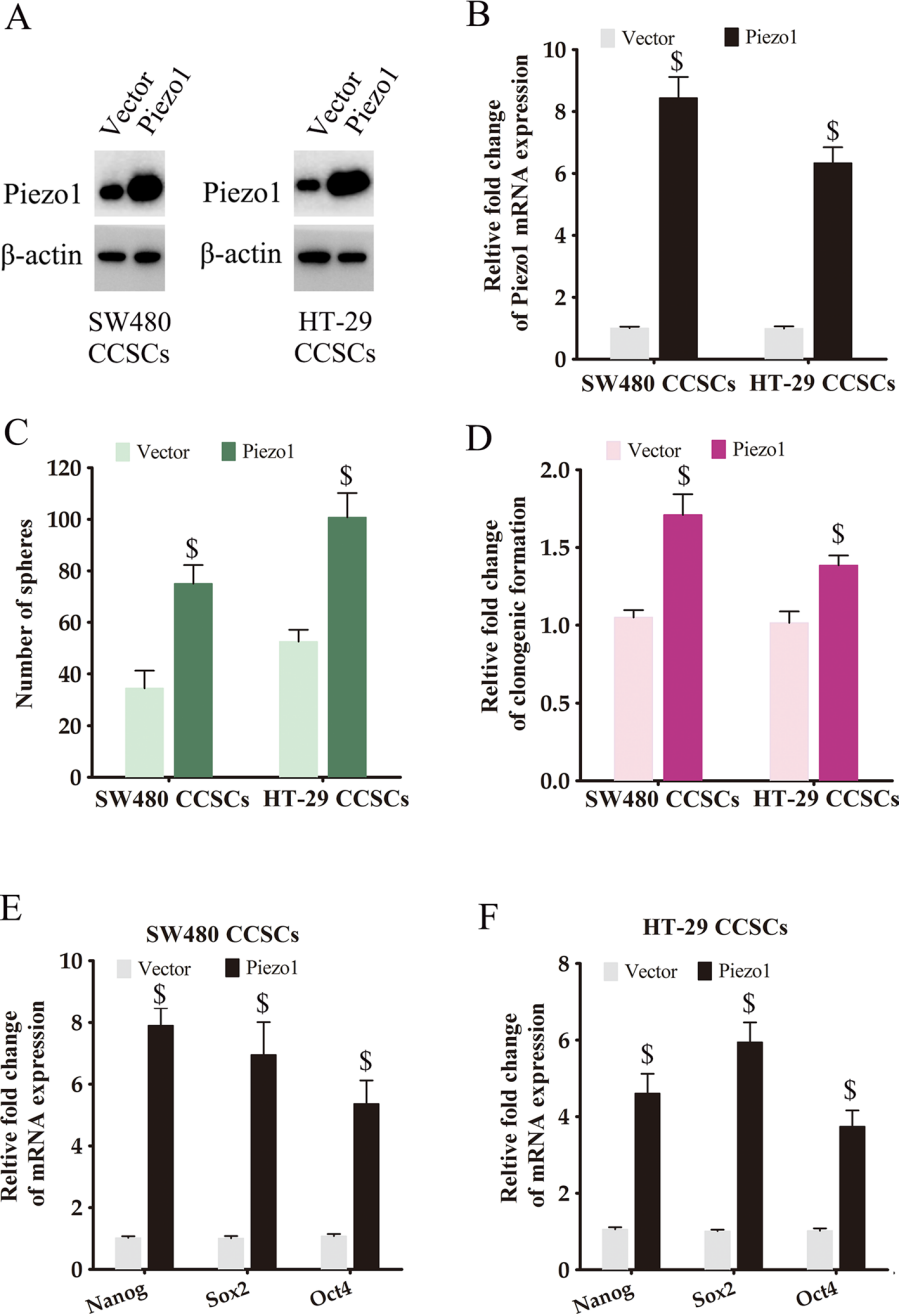
Piezo1 overexpression promoted stemness of CCSCs. **A**, **B** Piezo1 protein and RNA expression in Piezo1-overexpressing CCSCs. The data represent the mean ± SD. *p < 0.05, versus empty vector. **C**, **D** Number of spheres and colonies formed by CCSCs transfected with empty vector or Piezo1. The data represent the mean ± SD. *p < 0.05, versus empty vector. **E**, **F** Nanog, Sox2 and Oct4 mRNA levels in CCSCs transfected with empty vector or Piezo1. The data represent the mean ± SD. *p < 0.05, versus empty vector

To further explore the role of Piezo1 in controlling the phenotype of CCSCs, we generated Piezo1-overexpressing SW480 CCSCs and HT-29 CCSCs by transfecting the cells with full-length human Piezo1 sequence. Increased Piezo1 protein and RNA expression was confirmed in the CCSCs (Fig. 4A, B). Furthermore, the Piezo1-overexpressing CCSCs showed enhanced self-renewal capacity compared to CCSCs transfected with the empty vector (Fig. 4C). Piezo1 overexpression also increased the colony formation capacity of the CCSCs (Fig. 4D). Furthermore, the stemness-related genes Nanog, Sox2 and Oct4 were upregulated in the Piezo1-overexpressing CCSCs (Fig. 4E, F). These results suggested that Piezo1 is critical in maintaining the stemness of CCSCs.

### Piezo1 maintains the tumorigenic potential and stemness of CCSCs via Ca^2+^/NFAT1 signaling pathway

**Fig. 5:**
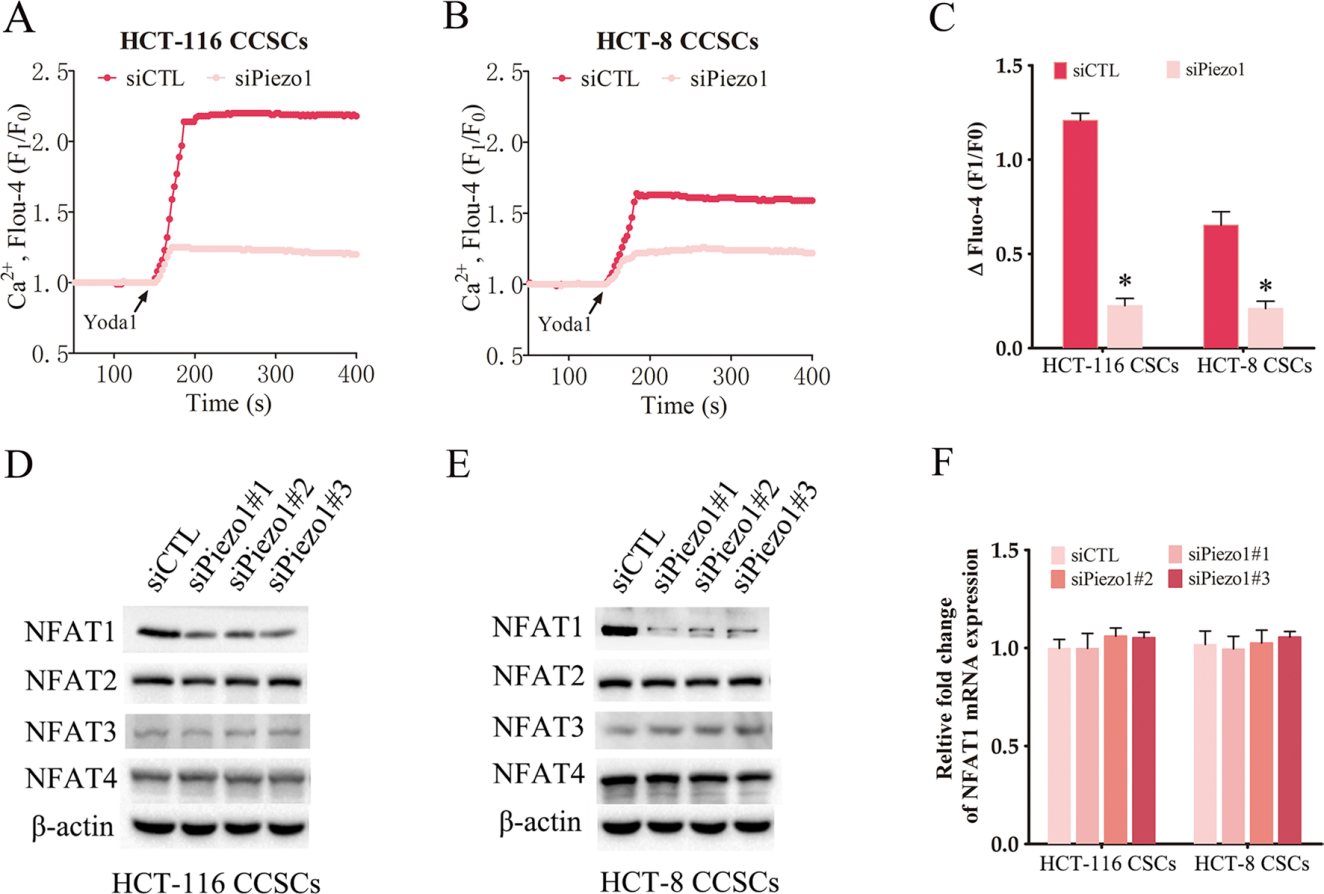
Piezo1 knockdown inhibited Ca^2+^/NFAT1 signaling in CCSCs. **A**–**C** Representative images and fluorescence intensity of intracellular Ca^2+^ in CCSCs transfected with siPiezo1 or siCTL. The data represent the mean ± SD. *p < 0.05, versus siCTL. **D**, **E** Immunoblot showing NFAT1, NFAT2, NFAT3 and NFAT4 protein expression in Piezo1-knockdown CCSCs. **F** NFAT1, NFAT2, NFAT3 and NFAT4 mRNA levels in the Piezo1-knockdown CCSCs. The data represent the mean ± SD. *p < 0.05, versus siCTL .

**Fig. 6:**
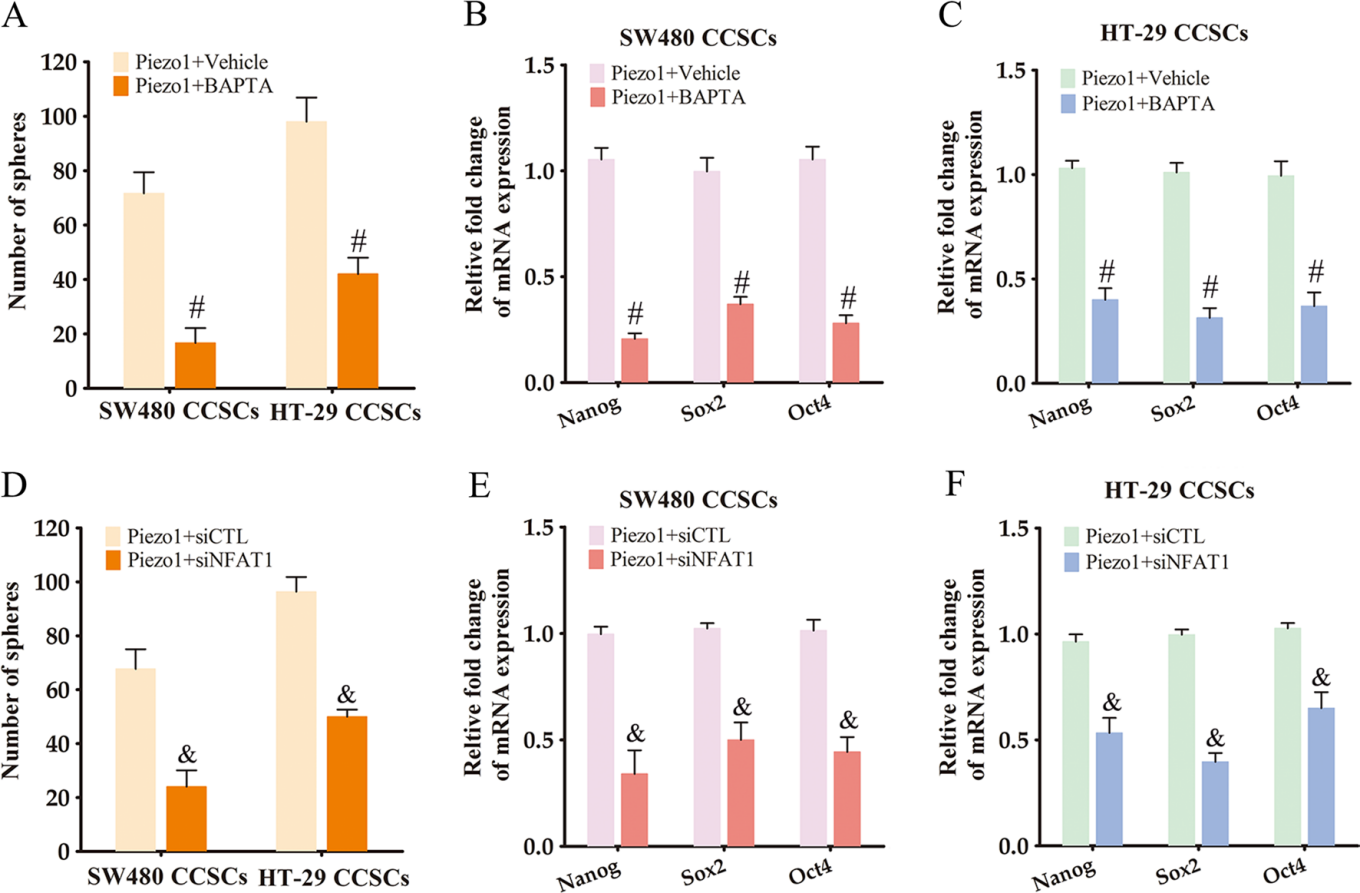
Piezo1 maintains the phenotype of CCSCs via Ca^2+^/NFAT1 signaling. **A** Number of spheres formed by Piezo1-overexpressing CCSCs treated with BAPTA/AM. The data represent the mean ± SD. *p < 0.05, versus Piezo1 + Vehicle. **B**, **C** Nanog, Sox2 and Oct4 mRNA expression in Piezo1-overexpressing CCSCs treated with BAPTA/AM. The data represent the mean ± SD. *p < 0.05, versus Piezo1 + Vehicle. **D** Number of spheres formed by Piezo1-overexpressing CCSCs transfected with siNFAT1 or siCTL. The data represent the mean ± SD. *p < 0.05, versus Piezo1 + siCTL. **E**, **F** Nanog, Sox2 and Oct4 mRNA expression in Piezo1-overexpressing CCSCs transfected with siNFAT1 or siCTL. The data represent the mean ± SD. *p < 0.05, versus Piezo1 + siCTL

To explore the mechanism underlying the role of Piezo1 in controlling the stemness of CCSCs, we analyzed Piezo1-mediated Ca^2+^ influx in the cells. The Piezo1 agonist Yoda1 rapidly increased intracellular Ca^2+^ levels in the CCSCs (Fig. 5A). However, Piezo1 knockdown in the CCSCs led to a decrease in Yoda1-induced Ca^2+^ influx (Fig. 5A, B). Piezo1-mediated Ca^2+^ influx can activate NFAT signaling [24], which is known to promote cancer stem cell populations and stemness features [25]. Consistent with this, NFAT1 protein expression was significantly decreased in the Piezo1-knockdown CCSCs, while NFAT2, NFAT3 and NFAT4 levels were unaffected (Fig. 5D, E). Moreover, the expression of NFAT1, NFAT2, NFAT3 and NFAT4 mRNAs were also not affected by knocking down Piezo1 (Fig. 5F). Thus, the reduction in NFAT1 protein expression was not due to transcriptional downregulation. In addition, pre-treatment with the intracellular Ca^2+^ scavenger BAPTA/AM reduced sphere formation by the Piezo1-overexpressing CCSCs (Fig. 6A), which coincided with decreased expression of the stemness-related genes (Fig. 6B, C). Consistent with the above findings, knocking down NFAT1 attenuated the sphere formation ability of Piezo1-overexpressing CCSCs (Fig. 6D). NFAT1 silencing also decreased the expression of Nanog, Sox2 and Oct4 mRNAs in the Piezo1-overexpressing CCSCs (Fig. 6E, F). Taken together, our results indicate that Piezo1 maintains the phenotype of CSCCs via the Ca^2+^/NFAT1 signaling pathway.

### Piezo1 regulates degradation of NFAT1 in CCSCs

**Fig. 7:**
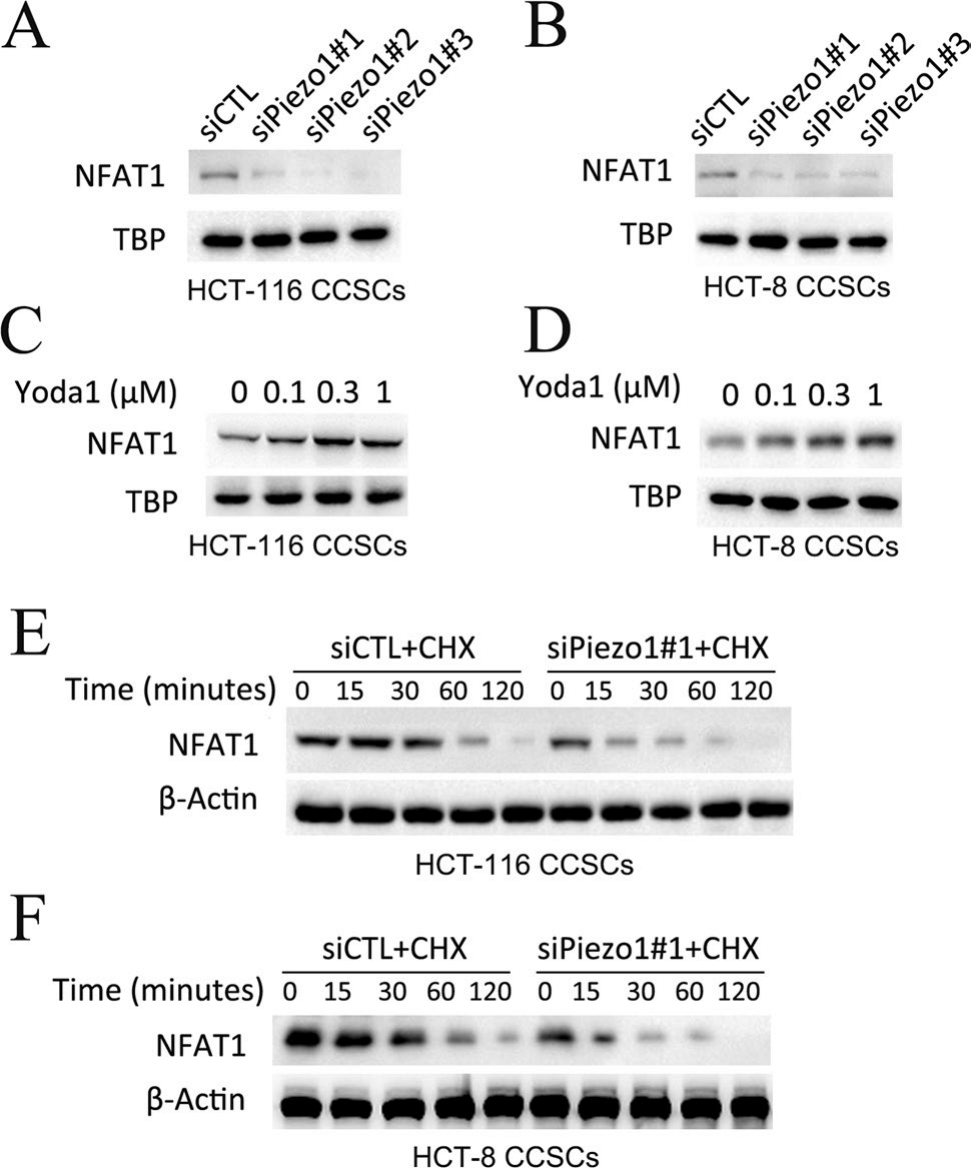
Piezo1 regulates degradation of NFAT1 in CCSCs. **A**, **B** Immunoblot showing NFAT1 and TBP protein expression in Piezo1-knockdown CCSCs. **C**, **D** Immunoblot showing NFAT1 and TBP protein expression in CCSCs treated with Yoda1. **E**, **F** CHX pulse-chase analysis of NFAT1 protein stability in Piezo1-knockdown CCSCs

We further validated the role of Piezo1 in NFAT1 activation by detecting the nuclear expression of NFAT1 in CCSCs. Piezo1 knockdown reduced the nuclear accumulation of NFAT1 in the SW480 CCSCs and HT-29 CCSCs (Fig. 7A, B). Interestingly, the accumulation of NFAT1 in the nuclei increased in CCSCs pre-treated with Yoda1 in a concentration-dependent manner (Fig. 7C, D). Since the inhibitory effect of Piezo1 knockdown on NFAT1 expression was likely exerted at the post-transcriptional level, we tested NFAT1 protein stability by treating the cells with the protein synthesis inhibitor cycloheximide (CHX). As expected, Piezo1 knockdown markedly reduced NFAT1 expression in the CHX-treated CCSCs compared to the CHX + siCTL group (Fig. 7E, F). Taken together, Piezo1 knockdown inhibits NFAT1 activation in the CCSCs by inducing the degradation of NFAT1 protein.

## Discussion

CCSCs are considered the “seed” cells of tumor growth and differentiation. Thus, it is essential to understand the mechanisms underlying the stemness of CCSCs in order to improve the prognosis of colon cancer. In the present study, we found that Piezo1 expression was high in the CD133^+^CD44^+^ CCSC fraction, and the Piezo1^high^/CD133^+^CD44^+^ colon cancer patients were in the advanced clinical stage. Knocking down Piezo1 in the CCSCs suppressed their stem-like characteristics via the Ca^2+^/NFAT1 signaling pathway. Furthermore, our findings indicate that Piezo1 may promote the degradation of NFAT1 protein through the proteasome pathway. Therefore, Piezo1 is a potential target for the selective elimination of CCSCs in colon cancer treatment.

There is emerging evidence that CCSCs control the genesis, relapse, malignancy and chemo-resistance of colon tumors [26, 27]. CD133 and CD44 are the characteristic surface markers of CCSCs. Yan et al. reported that the CD133^+^CD44^+^ fraction of HCT-8 cells are more resistant to doxorubicin treatment [19]. Furthermore, Botchkina et al. isolated the CD133 + CD44 + fraction of various colon cancer cell lines, and found that these cells exhibited stemness features [23]. Chen et al. also showed that CD133^+^CD44^+^ HCT-116 cells had significant invasive and self-renewal capacity in vivo and in vitro [28]. We found that Piezo1 was highly expressed in the CD133^+^CD44^+^ colon cancer tissues, and patients with Piezo1^high^/CD133^+^CD44^+^ tumors were usually in the advanced stage. Consistent with our findings, Sun et al. reported that Piezo1 is overexpressed in colon cancer patients and correlates with poor prognosis [15]. Piezo1 expression and function are altered in multiple cancers. For instance, Piezo1 is expressed at higher levels in the squamous cell carcinoma [28], bladder cancer [29], prostate cancer [14] and breast cancer tissues [30] compared to the corresponding normal tissues, indicating that it may be involved in cancer progression. In addition, the periosteal stem cells also express higher levels of Piezo1 [17], and Piezo1 also maintains the characteristic features of neural stem cells [18]. In line with this, we detected higher expression of Piezo1 in the CD133^+^CD44^+^ CCSC fraction isolated from different colon cancer cell lines. Silencing Piezo1 decreased the tumorigenic and self-renewal capacity of CCSC in vitro and in vivo, whereas overexpression of Piezo1 enhanced clonogenic potential, and stemness, and increased the expression of stemness-related genes. These findings suggested that Piezo1 may be critical in controlling the phenotype of CCSCs.

Intracellular calcium is essential for the maintenance of cancer stem cells. Blocking calcium release from the endoplasmic reticulum (ER) diminished the breast cancer stem cell population [31]. Furthermore, inhibition of calcium influx decreased the number and stemness of oral cancer stem cells [32]. Recently, Vladislav et al. showed that Piezo1-induced calcium influx may contribute to the migratory activity of human endometrial mesenchymal stem cells [16]. In this study, we found that Piezo1 knockdown suppressed Yoda1-induced calcium flux in the CCSCs, and calcium scavenging by BAPTA/AM neutralized the increased stemness of the Piezo1-overexpressing CCSCs. Nuclear factor of activated T cells (NFAT) is the main downstream target of calcium signaling and plays a key role in regulating cancer stemness. We found that NFAT1 protein level was significantly decreased in the Piezo1-knockdown CCSCs, while that of NFAT2, NFAT3 and NFAT4 were unaffected. However, Piezo1 knockdown did not affect NFAT1 RNA expression in the CCSCs. Interestingly, Piezo1 knockdown reduced the nuclear accumulation of NFAT1 in the CCSCs, whereas its overexpression increased NFAT1 levels in the nuclear fraction. Moreover, NFAT1 downregulation reduced Piezo1-induced sphere formation and upregulation of stemness-related genes. Consistent with our findings, Lang et al. reported that NFATc2, also known as NFAT1, promotes stem cell-like properties of CCSCs [33]. In addition, NFAT1 enhanced the self-renewal, invasion, tumorigenesis, and chemo-resistance of tumor-initiating lung adenocarcinoma cells [34]. We found that Piezo1 knockdown decreased the stability of NFAT1 protein in the CCSCs pre-treated with the protein synthesis inhibitor CHX. These findings suggested that Piezo1 knockdown repressed the stemness of CCSCs through inhibition of Ca^2+^/NFAT1 signaling and deregulation of NFAT1. However, further studies are needed to explore the mechanism of NFAT1 deregulation.

In conclusion, Piezo1 maintains the tumorigenicity and stemness of CCSCs via Ca^2+^/NFAT1 signaling. Piezo1^high^/CD133^+^CD44^+^ colon tumors are associated with advanced clinical stage, suggesting that the presence of Piezo1^high^/CD133^+^CD44^+^ CCSCs is a potential prognostic marker, and Piezo1 is a promising target for the selective elimination of CCSCs.

## Supplementary Information

Below is the link to the electronic supplementary material.

Supplementary file1 (DOCX 163 KB)

## Data Availability

The datasets generated during and/or analysed during the current study are available from the corresponding author on reasonable request.
