# Supplementary material for: Identification of Piezo1 as a potential target for therapy of colon cancer stem-like cells
Source: Discov Oncol. 2023 Jun 12;14:95. doi: 10.1007/s12672-023-00712-4 (PMC10260724; doi:10.1007/s12672-023-00712-4)
Supplement: Supplementary file 1 — Supplementary file1 (DOCX 163 KB) [file 12672_2023_712_MOESM1_ESM.docx]

**Supplementary Figures**

**
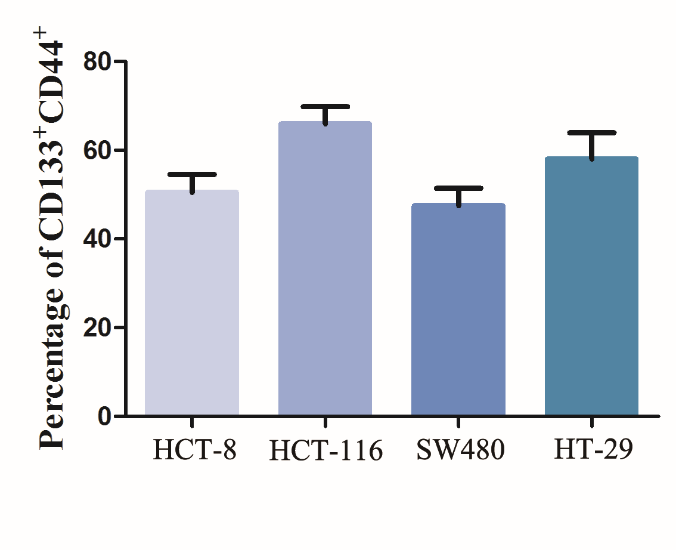
**

Supplementary Figure 1. The percentage of CD133^+^CD44^+^ fraction isolated from HCT-8, HCT-116, SW480 and HT-29 cell lines. The data represent the mean ± SD.


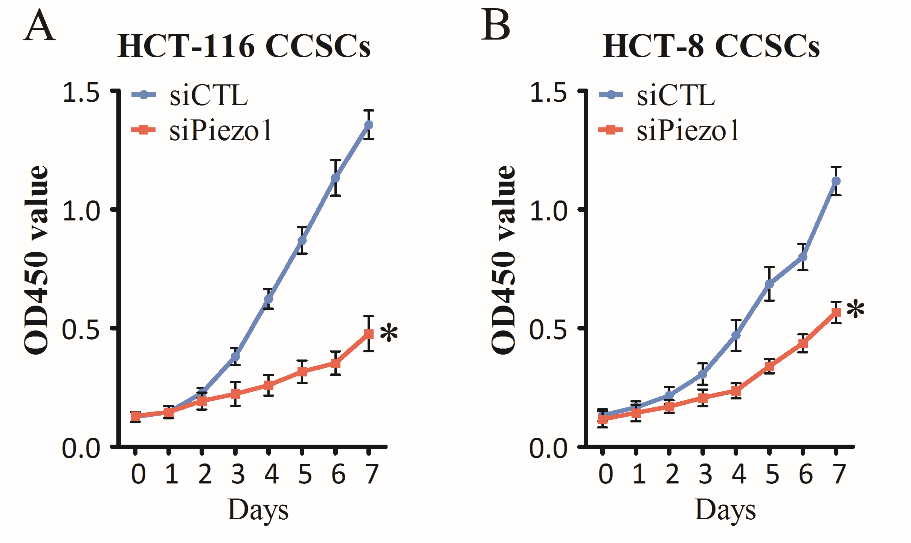


Supplementary Figure 2. A and B: Cell growth of CCSCs from HCT-116 and HCT-8 cell lines after transfection with siPiezo1 or siCTL. The data represent the mean ± SD. * p<0.05, versus siCTL.
